# Collaborative Multi-Robot Transportation in Obstacle-Cluttered Environments via Implicit Communication

**DOI:** 10.3389/frobt.2018.00090

**Published:** 2018-08-07

**Authors:** Charalampos P. Bechlioulis, Kostas J. Kyriakopoulos

**Affiliations:** Mechanical Engineering, National Technical University of Athens, Athens, Greece

**Keywords:** cooperative manipulation, implicit communication, interaction forces, obstacle avoidance, prescribed performance estimator

## Abstract

This paper addresses the problem of cooperative object transportation in a constrained workspace involving static obstacles, with the coordination relying on implicit communication established via the commonly grasped object. In particular, we consider a decentralized leader-follower architecture for multiple mobile manipulators, where the leading robot, which has exclusive knowledge of both the object's desired configuration and the position of the obstacles in the workspace, tries to navigate the overall formation to the desired configuration while at the same time it avoids collisions with the obstacles. On the other hand, the followers estimate the object's desired trajectory profile via novel prescribed performance estimation laws that drive the estimation errors to an arbitrarily small predefined residual set. Moreover, a navigation function-based scheme is innovatively combined with adaptive control to deal with parametric uncertainty. Hence, the current state of the art in robust motion planning and collision avoidance is extended by studying second order non-linear dynamics with parametric uncertainty. Furthermore, the feedback relies exclusively on each robot's force/torque, position as well as velocity measurements and no explicit information is exchanged online among the robots, thus reducing the required communication bandwidth and increasing robustness. Finally, two simulation studies clarify the proposed methodology and verify its efficiency.

## 1. Introduction

**Figure 1:**
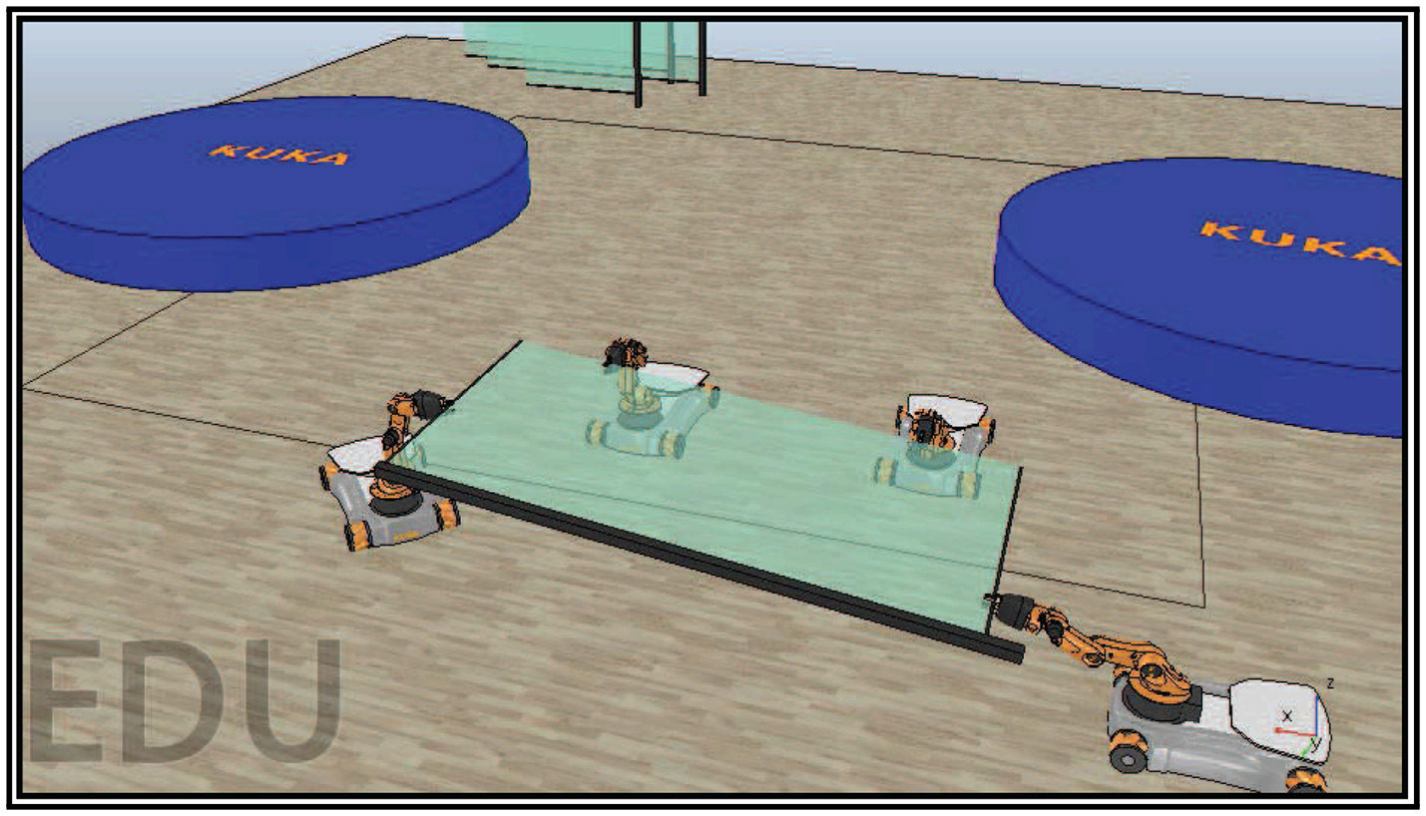
Four mobile manipulators handling a rigidly grasped object in a constrained workspace with static obstacles.

The recent development of robotic technologies has introduced robots in various fields of industry, agriculture, security, etc. However, complex applications require multiple robots to execute a task in coordination efficiently, e.g., handling a heavy object (see Figure 1) or assembling a complex product. Thus, a great research effort has been made during the last three decades on the coordinated control of multiple robots.

Most of the seminal works in this direction proposed centralized control algorithms, based on global information with respect to a common coordinate system. Particularly, centralized control systems are effective in the coordinated motion control of fixed-base manipulators, since the number of robots in coordination is usually limited to two or three. However, the recent advances in mobile manipulators, which allow free motion in a real world environment, have substantially increased the number of robots that can be involved in a coordinated task. Thus, centralized approaches render unrealistic, owing to the computational burden and the fact that various geometric errors that appear inevitably among the robots cannot be handled accurately based on a common coordinate system. To overcome the aforementioned issues, decentralized control of multiple robots emerged, in which each robot is controlled by its own controller based on its own local coordinate system.

**Figure 2:**
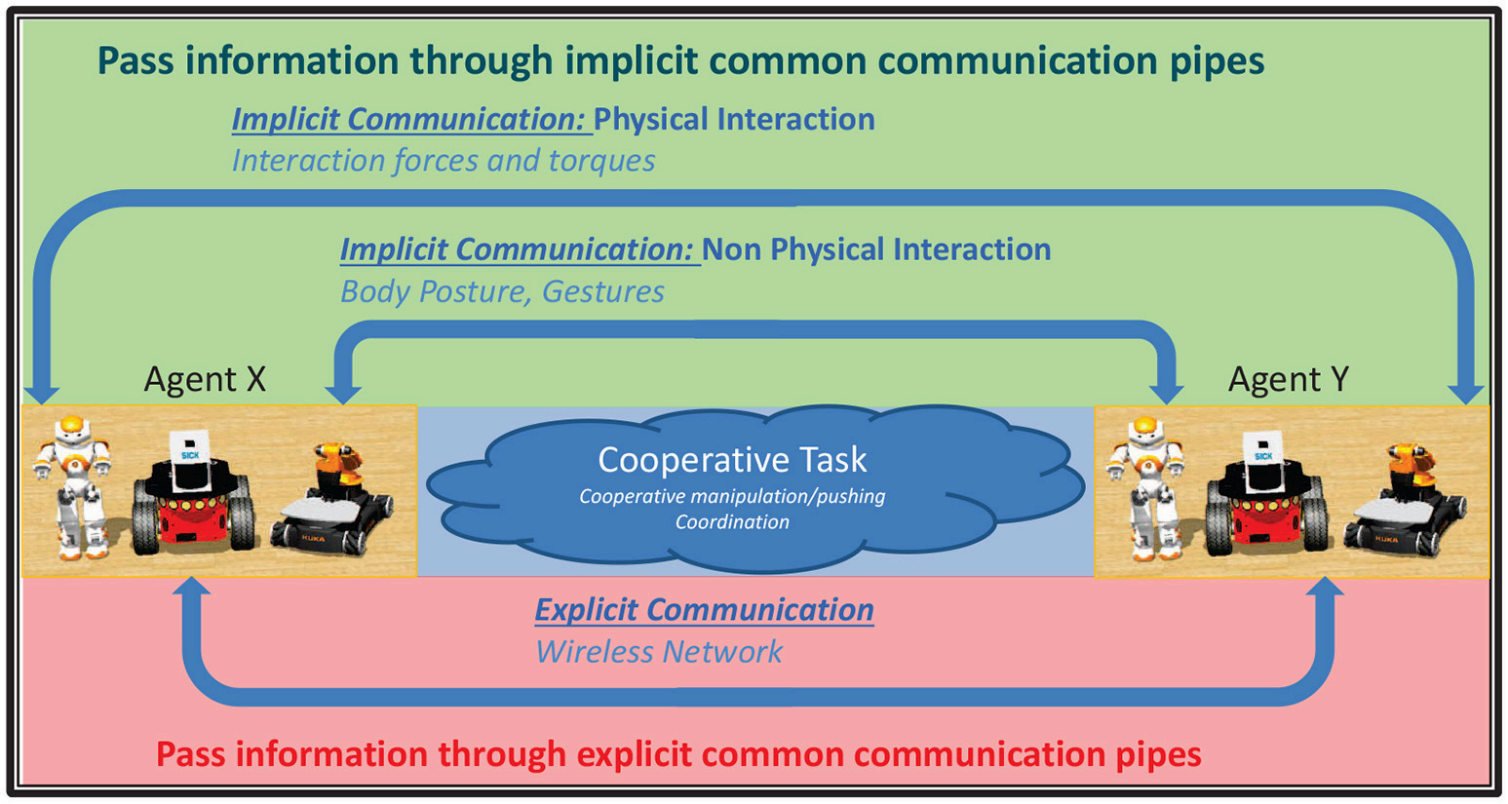
The two types of communication, namely the explicit and the implicit.

The study of decentralized multi-robot systems in object manipulation tasks has received increasing consideration over the last two decades. In particular, the communication among the robots has been proven critical, since no central unit exists to supervize the robots' actions. In general, two types of communication occur, namely the explicit and the implicit (see Figure 2). The former type is designed solely to convey information such as control signals or sensory data directly to other robots (Pereira et al., 2002). On the other hand, the latter occurs as a side-effect of robot's interactions with the environment or other robots, either physically (e.g., via the interaction forces between the object and the robot) or non-physically (e.g., via visual observation) and in such case, the information needed is acquired by appropriately installed sensors.

The most studied and frequently adopted communication form is the explicit one. It usually leads to simpler mathematical analysis and renders teams more effective. However, in case of communication environments that are prone to faults, severe problems may arise, such as object dropping, appliance of excessive forces and performance downgrading. Moreover, as the number of cooperating robots increases, complex design of communication networks is required to deal with crowded bandwidth (Stilwell and Bishop, 2000). On the other hand, the aforementioned limitations can be surpassed by employing implicit communication instead. Despite the complexity of the mathematical analysis, it yields simpler protocols and saves bandwidth as well as power, since no or very few data is explicitly exchanged. Furthermore, robustness is increased significantly in case of communication environments that are prone to failures. Moreover, although explicit communication leads, when accurately employed, to superior performance, nonetheless it is not essential for certain tasks when the implicit form is available. It should also be noted that more complicated communication protocols may offer little or no benefit over implicit communication (Balch and Arkin, 1994; Donald, 1995).

Cooperative manipulation has been well-studied in the literature, especially the centralized schemes (e.g., Uchiyama and Dauchez, 1988; Khatib, 1988; Schneider and Cannon, 1992; Tanner et al., 2003). In Uchiyama and Dauchez (1988) a hybrid position/force control is presented. In Khatib (1988), the overall closed-chain system is treated as an augmented object, with its inertial properties expressed via a single inertia matrix. Tanner et al. (2003) propose a centralized motion planning methodology for non-holonomic mobile manipulators, handling a deformable object. Navigation is based on dipolar inverse Lyapunov functions with guaranteed collision avoidance and convergence to the goal. The concept of object impedance control is presented in Schneider and Cannon (1992). An impedance law specifies the relation between the object's accelerations, external forces and kinematic state. Nevertheless, despite its efficacy, centralized control is less robust, since all robots depend on a central system and its complexity rises sharply as the number of team-robots increases. On the other hand, decentralized control usually depends on either explicit communication or off-line knowledge of the desired object trajectory, (e.g., Khatib et al., 1996; Dickson et al., 1997; Liu et al., 1996). Furthermore, position-force hybrid control schemes, where the position of the object is controlled toward a given direction in the workspace and the internal forces on the object are controlled close to the origin are presented in Zhang et al. (2016), Petitti et al. (2016), and Noohi and Zefran (2016). Moreover, in other leader-follower schemes (e.g., Luh and Zheng, 1987; Sugar and Kumar, 1998), the leader has to transmit on-line the desired object trajectory to the follower.

Alternatively, implicit communication has been adopted in various decentralized studies on mobile manipulators. Kosuge's research group in a series of works (e.g., Kosuge and Oosumi, 1996; Kosuge et al., 1997a,b), presented a leader-follower scheme for holonomic manipulators in free space. The leader implements a desired trajectory profile through an impedance scheme, while the follower estimates it through the motion of the object. However, the estimation error remains bounded close to zero only if the desired acceleration is zero (i.e., trajectories with constant velocity profile). Regarding non-holonomic mobile robots, the follower's passive caster behavior was adopted in Stilwell and Bay (1993) and Kosuge et al. (1998). Although, the stability of the follower's contact is established, it is not mentioned how the object's trajectory can be controlled. Alternatively, Gross et al. (2006a,b) and Gross and Dorigo (2008) designed a motion coordination controller with no explicit communication for a group of physically connected robots using only interaction force measurements. In a similar direction but following a pushing-only strategy, Chen et al. (2013, 2015) employed a visual occlusion notion to guide the robot swarm to the goal position without exchanging any information. Finally, another algorithm that does not require any explicit communication network among the robots, but instead, the robots coordinate their actions through sensing the motion of the object itself was presented in a series of recent works in Wang and Schwager (2016a,b,c, 2015).

This paper extends our recent results in Tsiamis et al. (2015a,b) by considering multiple mobile manipulators in the problem of decentralized cooperative object manipulation in a constrained workspace with static obstacles. The challenge lies in completely displacing explicit communication with implicit, which results naturally from the physical interaction of the robots via the object (i.e., as the robots move, forces and torques are exerted in certain directions at the robot/object contacts). Similarly to Tsiamis et al. (2015b), the considered architecture is a leader-follower scheme. The leader is aware of both the object's desired configuration as well as of the position of the obstacles in the workspace and employs a navigation function based scheme to calculate the object's desired trajectory and avoid collisions with the obstacles and the workspace boundary. The followers, without knowing a priori the object's goal trajectory, estimate it by observing its motion. The estimation process is based on the prescribed performance methodology (Bechlioulis and Rovithakis, 2010), which drives the estimation error to an arbitrarily small residual set. Moreover, the robots employ adaptive laws to compensate for the parametric uncertainty of their dynamic models. Finally, it should be noticed that all robots use solely their own force/torque, position and velocity measurements, thus avoiding any inter-robot explicit communication.

In this work, navigation functions (Koditschek and Rimon, 1990) are also innovatively combined with adaptive control to deal with the parametric uncertainty in the robot dynamics. Hence, we extend the current state of the art in robust motion planning by studying second order non-linear dynamics with parametric uncertainty. We also extend the current state of art in implicit communication-based cooperative manipulation (Kosuge and Oosumi, 1996; Kosuge et al., 1997a,b), via a more robust estimation algorithm that converges even though the desired object's acceleration profile is non-zero (i.e., arbitrary object's desired trajectory profile as long as it is bounded and smooth). Moreover, the customizable ultimate bounds allow us to achieve practical stabilization of the estimation error, with accuracy limited only by the sensors' resolution.

The rest of the manuscript is organized as follows: section 2 introduces the problem and describes the model of the system. The control methodology along with the estimation algorithm are presented in section 3. Section 4 validates our approach via two simulated paradigms. Finally, section 5 concludes the paper and discusses future research directions.

## 2. Problem formulation

In conventional coordinated manipulation control problems, a task is generally characterized by the desired motion of the handled object. However, in decentralized multi-robot systems, since there is no common coordinate system owing to the fact that each robot should be controlled on its own local coordinate system, the task cannot be parameterized in a conventional way. Hence, ordinarily we select a robot as a leader and we define its coordinate system as a reference one to describe the desired object motion based on it. For the rest of the robots, referred to as followers, their task is described by the relative motion with respect to the commonly grasped object. In this way, the motion of the object is determined by the motion of the leader and the task is specified by the motion of the followers. Consequently, since the task of the followers is described by the relative motion with respect to the commonly grasped object, which is controlled on the local coordinate system based on local sensory information, the followers should observe/estimate the motion of the manipulated object.

In this work, we consider *N* + 1 mobile manipulators under a leader-follower architecture, handling a rigidly grasped object in a constrained workspace Q with static obstacles. We assume that each robot has at least 6 DoFs and is fully actuated at its end-effector (i.e., any force and torque along and around all axis of the end-effector's frame can be applied). It should also be noted that only the leading robot is aware of the obstacles' position in the workspace and the object's desired configuration $P_{O}^{d}\in Q$, whereas the followers should estimate the object's desired trajectory profile and manipulate the object in coordination with the leader based solely on their own local sensory information. In this respect, we assume that measurements of position, velocity and interaction forces/torques with respect to the object are available for each robot exclusively. Additionally, the geometric parameters of the mobile manipulators are considered known, whereas their dynamic parameters are completely unknown. Moreover, the control for each robot will be designed based on a commonly agreed frame on the object. Nevertheless, it should be stressed that such assumption is realistic since each robot could identify the common frame on the object employing a visual detection system with respect to a feature on the object. Finally, notice that we have not considered other types of implicit communication, for instance via cameras or other line of sight devices like range finders and laser scanners, which work effectively already in certain multi-robot systems in non-physical coordination. However, in a transportation task it should be noted that the commonly handled object raises severe occlusion issues that would probably block the line of sight sensing devices. In this respect, certain other secondary tasks, such as connectivity maintenance, should be considered in parallel, increasing however the complexity of the approach.

### 2.1. Kinematics

We denote the coordinates of the commonly agreed frame on the object as well as the leader's and followers' task-space (i.e., end-effector) coordinates with respect to an inertial frame {*I*}^1^ by:

(1) $$\begin{array}{l} P_{O}≜{\left[\eta_{1,O}^{T},\eta_{2,O}^{T}\right]}^{T},\;\;P_{L}≜{\left[\eta_{1,L}^{T},\eta_{2,L}^{T}\right]}^{T},\\ \;P_{F_{i}}≜{\left[\eta_{1,F_{i}}^{T},\eta_{2,F_{i}}^{T}\right]}^{T},\;i=1,\ldots ,N \end{array}$$

where $\eta_{1,j}≜{\left[x_{j},y_{j},z_{j}\right]}^{T}$ and $\eta_{2},j≜{\left[\phi_{j},\theta_{j},\psi_{j}\right]}^{T}$, *j* ∈ {*O, L, F*_1_, …, *F*_*N*_} correspond to the position and orientation, expressed in the Euler angles representation, with respect to the inertial frame. Since the common object frame is identified by the onboard sensory information (e.g., a local visual tracking system), each robot may easily compute the object's coordinates with respect to the inertial frame via a fixed transformation, without requesting any information via external communication with a global tracking system. Furthermore, owing to the fact that the object is rigidly grasped, we establish a velocity relation as follows:

(2) $${\dot{P}}_{L}=J_{LO}{\dot{P}}_{O},\;{\dot{P}}_{F_{i}}=J_{F_{i}O}{\dot{P}}_{O},\;i=1,\ldots ,N$$

where *J*_*LO*_ and *J*_*F*_*i*_*O*_, *i* = 1, …, *N* denote the adjoint transformation matrix from the end-effector of each robot to the object's frame (Murray et al., 1994, p. 55). Notice that since the end-effector and the object are rigidly connected, the aforementioned transformation matrices have always full rank and hence obtain a well-defined inverse. Thus, each robot may calculate the object's velocity through (2).

### 2.2. Dynamics

The dynamic model in terms of task space coordinates is described by:

(3) $$M_{i}\left(P_{i}\right){\ddot{P}}_{i}+C_{i}\left(P_{i},{\dot{P}}_{i}\right){\dot{P}}_{i}+D_{i}\left(P_{i},{\dot{P}}_{i}\right)+G_{i}\left(P_{i}\right)=U_{i}+F_{i}$$

where *M*_*i*_(*P*_*i*_), *i* ∈ {*L, F*_1_, …, *F*_*N*_} denote the positive definite inertial matrices, *C*_*i*_(*P*_*i*_, *Ṗ*_*i*_), *i* ∈ {*L, F*_1_, …, *F*_*N*_} represent coriolis and centrifugal terms, *D*_*i*_(*P*_*i*_, *Ṗ*_*i*_), *i* ∈ {*L, F*_1_, …, *F*_*N*_} model joint friction effects and *G*_*i*_(*P*_*i*_), *i* ∈ {*L, F*_1_, …, *F*_*N*_} encapsulate gravitational forces and torques. Furthermore, the vectors ***F***_*i*_, *i* ∈ {*L, F*_1_, …, *F*_*N*_} represent the interaction forces and torques exerted at the end-effector by the object and *U*_*i*_, *i* ∈ {*L, F*_1_, …, *F*_*N*_} denote the task space control input wrenches. It is also known that uncertain physical parameters of the robots, such as link masses and inertias as well as joint friction coefficients, appear linearly in the robot dynamic model (3). Hence, we may express the dynamics in terms of a set of unknown but constant parameters $\theta_{i}\in {ℜ}^{Q_{i}}$, *i* ∈ {*L, F*_1_, …, *F*_*N*_} in the following way:

(4) $$M_{i}\left(a\right)d+C_{i}\left(a,b\right)c+D_{i}\left(a,b\right)+G_{i}\left(a\right)=Z_{i}^{T}\left(a,b,c,d\right)\theta_{i}$$

where *Z*_*i*_(*a, b, c, d*), *i* ∈ {*L, F*_1_, …, *F*_*N*_} are *Q*_*i*_ × 6 regressor matrices composed of known non-linear functions. Finally, a skew-symmetric property of the matrices *Ṁ*_*i*_(*P*_*i*_) − 2*C*_*i*_(*P*_*i*_, *Ṗ*_*i*_), *i* ∈ {*L, F*_1_, …, *F*_*N*_} is also fulfilled.

Remark 1. *The relation between the robot joint force/torque control input* τ_*i*_, *i* ∈ {*L, F*_1_, …, *F*_*N*_} *and the task space control input wrench U*_*i*_, *i* ∈ {*L, F*_1_, …, *F*_*N*_} *is given by:*

(5) $$\tau_{i}=J_{i}^{\#T}U_{i}+\left(I-J_{i}^{T}J_{i}^{\#T}\right)\tau_{i}^{0}\text{, }i\in \left\{L,F_{1},\ldots ,F_{N}\right\}$$

*where* $J_{i}^{\#}$*, i* ∈ {*L, F*_1_, …, *F*_*N*_} *denote the generalized inverse of the robot Jacobians, that is consistent with the equations of motion of the mobile manipulators' joints and their end-effectors (see Khatib, 1988). Notice that the vector* $\tau_{i}^{0}$ *does not contribute to the end-effector's wrench and thus can be regulated independently to achieve secondary goals (e.g., increase of velocity/force manipulability)*.

## 3. Methodology

The leader is aware of both the desired configuration of the object as well as of the obstacles' position in the workspace. Thus, its control objective is to navigate the overall formation toward the goal configuration while at the same time it avoids collisions with the static obstacles that lie within the workspace. Toward this direction, the concept of Navigation Functions in Koditschek and Rimon (1990) will be innovatively combined with adaptive control to deal with the parametric uncertainty in the robot dynamics (3). On the other hand, the followers are not aware of the object's trajectory. However, even though explicit inter-robot communication is not permitted, the followers will estimate the object's desired trajectory profile via their own state measurements. Toward this direction, acceleration residuals owing to the lack of acceleration measurements will be compensated by adopting a robust prescribed performance estimator that guarantees ultimate boundedness of the estimation errors with predefined transient and steady state specifications. Finally, an adaptive control scheme will be designed to achieve asymptotic tracking of the estimated trajectory profile, increasing thus the robustness of the overall control scheme and avoiding high interaction forces between the object and the robots.

### 3.1. Leader's control scheme

The control design relies on the Navigation Function concept originally proposed by Koditschek and Rimon (1990) as follows:

(6) $$\Phi_{O}(P_{O};P_{O}^{d})=\frac{\gamma (P_{O}-P_{O}^{d})}{{\left({\left(\gamma (P_{O}-P_{O}^{d})\right)}^{k}+\beta (P_{O})\right)}^{\frac{1}{k}}}$$

where *k* > 1 is a design constant, $\gamma :Q\to {\mathbb{R}}_{+}$ with γ(**0**) = 0 represents the attractive potential field to the goal configuration $P_{O}^{d}$ (e.g., $\gamma \left(P_{O}-P_{O}^{d}\right)≜||P_{O}-P_{O}^{d}|{|}^{2}$) and $\beta :Q\to {\mathbb{R}}_{+}$ with $\underset{P_{O}\to \{\begin{array}{l} \text{Boundary}\\ \text{Obstacles} \end{array}}{\lim}\beta \left(P_{O}\right)=0$ represents the repulsive potential field by the workspace boundary and the obstacle regions (e.g., $\beta \left(P_{O}\right)≜\Pi_{j=0}^{M}\beta_{j}\left(P_{O}\right)$, where β_*j*_(*P*_*O*_) denote appropriately selected distance functions to the workspace boundary for *j* = 0 and to the obstacle regions for *j* = 1, …, *M*). Without loss of generality^2^, we adopt spherical regions to model the robots, the object, the workspace and the obstacles. In that respect, it was proven in Koditschek and Rimon (1990) that $\Phi_{O}\left(P_{O},P_{O}^{d}\right)$ obtains a global minimum at $P_{O}^{d}$ and no other local minima for sufficiently large *k*. Thus, a feasible path that leads from any initial obstacle-free configuration^3^ to the desired configuration might be generated by following the negated gradient of $\Phi_{O}\left(P_{O},P_{O}^{d}\right)$. Consequently, the leader's end-effector desired motion profile is designed as follows:

(7) $${\dot{P}}_{L}^{d}\left(t\right)=-k_{NF}J_{LO}\nabla_{P_{O}}\Phi_{O}\left(P_{O}\left(t\right),P_{O}^{d}\right)\text{, }k_{NF}>0.$$

In the sequel, we propose an adaptive control scheme for the leader's end-effector that guarantees the asymptotic stabilization of the object to the goal configuration $P_{O}^{d}$.

**Theorem 1**. *Consider the unknown leader's dynamics (3) that obeys the parametric property (4), as well as the desired motion profile (7). The adaptive control scheme:*

(8) $$\begin{array}{l} U_{L}=-F_{L}+Z_{L}^{T}\left(P_{L},{\dot{P}}_{L},{\dot{P}}_{L}^{d},{\ddot{P}}_{L}^{d}\right){\hat{\theta }}_{L}-K_{L}S_{L}\\ \;-\frac{1}{1-\Phi \left(P_{O},P_{O}^{d}\right)}{J_{LO}}^{-T}\nabla_{P_{O}}\Phi \left(P_{O},P_{O}^{d}\right)\text{, }K_{L}>0\\ {\dot{\hat{\theta }}}_{L}=-\Gamma_{L}Z_{L}\left(P_{L},{\dot{P}}_{L},{\dot{P}}_{L}^{d},{\ddot{P}}_{L}^{d}\right)S_{L}\text{, }\Gamma_{L}>0\text{,} \end{array}$$

*where* $S_{L}\left(t\right)={Ṗ}_{L}\left(t\right)-{Ṗ}_{L}^{d}\left(t\right)$ *denotes the velocity error and* ${\hat{\theta }}_{L}$ *denotes the estimate of the unknown dynamic parameters* θ_*L*_ *of the leader's model, guarantees for a sufficiently large parameter k* > 1 *of the Navigation Function* $\Phi_{O}\left(P_{O},P_{O}^{d}\right)$ *defined in (6), that the object is asymptotically stabilized to* $P_{O}^{d}$ *except from a set of initial conditions of measure zero*.

*Proof:* Consider the positive definite function:

$$V_{L}=\ln\left(\frac{1}{1-\Phi_{O}\left(P_{O},P_{O}^{d}\right)}\right)+\frac{1}{2}S_{L}^{T}M_{L}\left(P_{L}\right)S_{L}+\frac{1}{2}{\tilde{\theta }}_{L}^{T}\Gamma_{L}^{-1}{\tilde{\theta }}_{L}$$

where *M*_*L*_(*P*_*L*_) is the positive definite inertial matrix and ${\tilde{\theta }}_{L}={\hat{\theta }}_{L}-\theta_{L}$ denotes the parametric error. Notice also that $\Phi_{O}\left(P_{O},P_{O}^{d}\right)$ takes values from the set [0, 1); hence the first term is well defined within the feasible workspace. Thus, differentiating *V*_*L*_ with respect to time and substituting (2) as well as the dynamics (3), we obtain:

$$\begin{array}{l} {\dot{V}}_{L}=\frac{1}{1-\Phi_{O}\left(P_{O},P_{O}^{d}\right)}\nabla_{P_{O}}^{T}\Phi_{O}\left(P_{O},P_{O}^{d}\right)J_{LO}^{-1}{\dot{P}}_{L}+S_{L}^{T}(U_{L}+F_{L}\\ -M_{L}\left(P_{L}\right){\ddot{P}}_{L}^{d}-C_{L}\left(P_{L},{\dot{P}}_{L}\right){\dot{P}}_{L}-D_{L}\left(P_{L},{\dot{P}}_{L}\right)-G_{L}\left(P_{L}\right))\\ \;+\frac{1}{2}S_{L}^{T}{\dot{M}}_{L}\left(P_{L}\right)S_{L}+{\tilde{\theta }}_{L}\Gamma_{L}^{-1}{\dot{\hat{\theta }}}_{L}\text{.} \end{array}$$

Adding and subtracting the terms $S_{L}^{T}C_{L}\left(P_{L},{Ṗ}_{L}\right){Ṗ}_{L}^{d}$ and $\frac{1}{1-\Phi_{O}\left(P_{O},P_{O}^{d}\right)}\nabla_{P_{O}}^{T}\Phi_{O}\left(P_{O},P_{O}^{d}\right)J_{LO}^{-1}{Ṗ}_{L}^{d}$ as well as substituting the desired motion profile (7), we get:

$$\begin{array}{l} {\dot{V}}_{L}=-\frac{k_{NF}{\left\|\nabla_{P_{O}}^{T}\Phi_{O}\left(P_{O},P_{O}^{d}\right)\right\|}^{2}}{1-\Phi_{O}\left(P_{O},P_{O}^{d}\right)}+S_{L}^{T}(U_{L}+F_{L}-M_{L}\left(P_{L}\right){\ddot{P}}_{L}^{d}\\ \;-C_{L}\left(P_{L},{\dot{P}}_{L}\right){\dot{P}}_{L}^{d}-D_{L}\left(P_{L},{\dot{P}}_{L}\right)-G_{L}\left(P_{L}\right)\;\\ \;+\frac{1}{1-\Phi_{O}\left(P_{O},P_{O}^{d}\right)}J_{LO}^{-T}\nabla_{P_{O}}\Phi_{O}\left(P_{O},P_{O}^{d}\right))+{\tilde{\theta }}_{L}\Gamma_{L}^{-1}{\dot{\hat{\theta }}}_{L}\;\\ \;+\frac{1}{2}S_{L}^{T}\left({\dot{M}}_{L}\left(P_{L}\right)-2C_{L}\left(P_{L},{\dot{P}}_{L}\right)\right)S_{L}\text{.} \end{array}$$

Thus, invoking the parametric property (4) as well as the skew-symmetry of *Ṁ*_*L*_(*P*_*L*_) − 2*C*_*L*_(*P*_*L*_, *Ṗ*_*L*_) we arrive at:

$$\begin{array}{l} {\dot{V}}_{L}=-\frac{k_{NF}{\left\|\nabla_{P_{O}}^{T}\Phi_{O}\left(P_{O},P_{O}^{d}\right)\right\|}^{2}}{1-\Phi_{O}\left(P_{O},P_{O}^{d}\right)}+S_{L}^{T}(U_{L}+F_{L}-Z_{L}^{T}\left(P_{L},{\dot{P}}_{L},{\dot{P}}_{L}^{d},{\ddot{P}}_{L}^{d}\right)\theta_{L}\\ \;+\frac{1}{1-\Phi_{O}\left(P_{O},P_{O}^{d}\right)}J_{LO}^{-T}\nabla_{P_{O}}\Phi_{O}\left(P_{O},P_{O}^{d}\right))+{\tilde{\theta }}_{L}\Gamma_{L}^{-1}{\dot{\hat{\theta }}}_{L}\text{.} \end{array}$$

Hence, substituting the control scheme (8) yields:

$${\dot{V}}_{L}=-\frac{k_{NF}{\left\|\nabla_{P_{O}}^{T}\Phi_{O}\left(P_{O},P_{O}^{d}\right)\right\|}^{2}}{1-\Phi_{O}\left(P_{O},P_{O}^{d}\right)}-S_{L}^{T}K_{L}S_{L}\leq 0.$$

Therefore, owing to the fact that $\frac{1}{1-\Phi_{O}\left(P_{O},P_{O}^{d}\right)}>1$ and invoking standard Lyapunov arguments (i.e., the function *V*_*L*_ is positive definite and its time derivative along the leader's dynamics is negative semi-definite) we may conclude the boundedness of *S*_*L*_, ${\tilde{\theta }}_{L}$, $\ln\left(\frac{1}{1-\Phi_{O}\left(P_{O},P_{O}^{d}\right)}\right)$ and consequently *P*_*O*_(*t*), *Ṗ*_*O*_(*t*), and ${Ṗ}_{L}^{d}\left(t\right)$. Hence, it can be easily deduced that $\Phi_{O}\left(P_{O},P_{O}^{d}\right)$ remains strictly less than 1, avoiding thus collisions with the obstacles and the workspace boundary. Furthermore, the uniform continuity of ${\dot{V}}_{L}$ can be easily inferred via calculating:

$${\ddot{V}}_{L}=-k_{NF}\frac{2\nabla_{P_{O}}^{T}\Phi_{O}\left(P_{O},P_{O}^{d}\right)\text{H}\Phi_{O}\left(P_{O},P_{O}^{d}\right)\left(1-\Phi_{O}\left(P_{O},P_{O}^{d}\right)\right)+{\left\|\nabla_{P_{O}}^{T}\Phi_{O}\left(P_{O},P_{O}^{d}\right)\right\|}^{2}\nabla_{P_{O}}^{T}\Phi_{O}\left(P_{O},P_{O}^{d}\right)}{{\left(1-\Phi_{O}\left(P_{O},P_{O}^{d}\right)\right)}^{2}}{\dot{P}}_{O}-2S_{L}^{T}K_{L}{\dot{S}}_{L}$$

with $\text{H}\Phi_{O}\left(P_{O},P_{O}^{d}\right)$ denoting the Hessian matrix of the Navigation Function, which is proven bounded owing to the boundedness of *Ṗ*_*O*_, $\ln\left(\frac{1}{1-\Phi_{O}\left(P_{O},P_{O}^{d}\right)}\right)$, *S*_*L*_ and *Ṡ*_*L*_^4^ that was established previously, as well as to the smoothness properties of the Navigation Function within the feasible workspace Q; thus invoking Barbalat's Lemma we conclude that $\lim_{t\to \infty }{\dot{V}}_{L}\left(t\right)=0$, from which we can easily deduce that $\lim_{t\to \infty }\|\nabla_{P_{O}}\Phi_{O}\left(P_{O}\left(t\right),P_{O}^{d}\right)\|=0$ and consequently that $\lim_{t\to \infty }\|{Ṗ}_{O}\left(t)\right\|=0$ since *Ṗ*_*O*_(*t*) was proven uniformly bounded. Finally, even though $\|\nabla_{P_{O}}\Phi_{O}\left(P_{O},P_{O}^{d})\right\|=0$ occurs at either the goal configuration $P_{O}^{d}$ or at a saddle point, all saddle points of $\Phi_{O}\left(P_{O},P_{O}^{d}\right)$ become isolated (Koditschek and Rimon, 1990) for sufficiently large *k* > 1 and hence, the set of initial conditions that lead to them are sets of measure zero (Milnor, 1963). As a result, the proposed control scheme guarantees the asymptotic stabilization of the object to the desired configuration $P_{O}^{d}$, except from a set of initial conditions of measure zero, which completes the proof.

**Figure 3:**
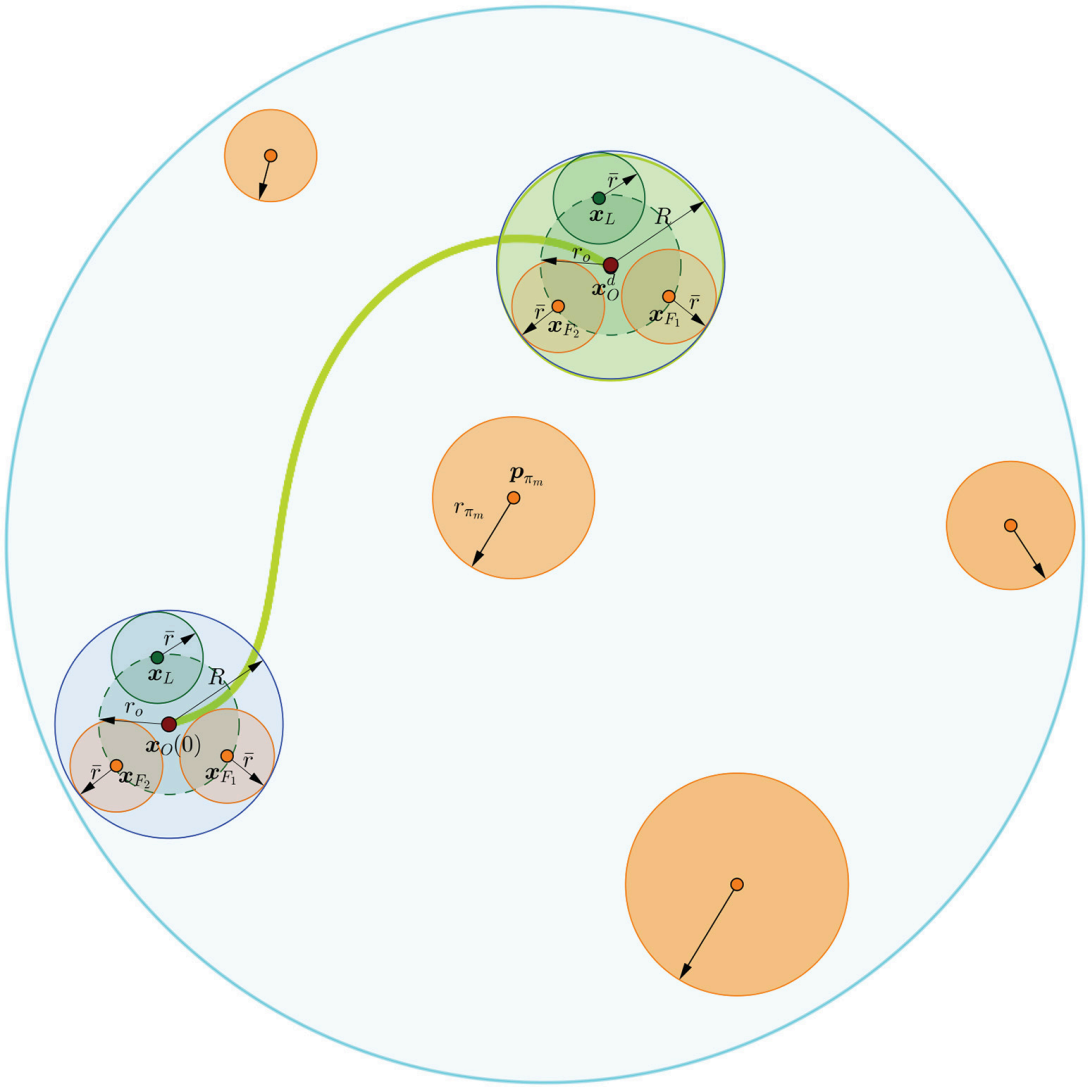
Graphical representation of a safe trajectory of the robotic team. The orange areas indicate the obstacles and the cyan line the workspace boundary. The blue line encircles the area covered by the robotic team and the object.

Remark 2. *Let us denote* $B\left(P_{O};r_{O}\right)$ *as the closed ball centered at *P*_*O*_ that includes the volume of the object and has radius *r*_*O*_. Let us also define the closed balls* $B\left(P_{i};r_{i}\right)$ *with radii* *r*_*i*_, *i* ∈ {*L, F*_1_, …, *F*_*N*_}*, centered at the end-effector of each robot, that cover the robot volume for all possible configurations. We also assume that the distance among the grasping points on the given object is at least r*_*i*_ + *r*_*j*_, ∀*i* ≠ *j* ∈ {*L, F*_1_, …, *F*_*N*_} *such that any pair of robots do not collide. Therefore, if we define the ball area* $B\left(P_{O};R\right)$ *centered at the origin of the object's body frame and comprises all aforementioned ball regions of the robotic team and the object (see Figure 3), then the problem at hand is recast into augmenting the workspace boundary by the radius *R* and considering the overall robotic team as a point, such that the Navigation Function strategy can be employed to guarantee the safe execution of the transportation task*.

Remark 3. *Artificial Potential Fields have been employed extensively in the past to deal with the robot navigation problem in both single and multi-agent formulations. However, single and double integrator models have been mostly studied so far without considering any robustness issues against model uncertainties (Mellinger and Kumar, 2010; Duan et al., 2013). In this work, Navigation Functions were innovatively combined with adaptive control to deal with parametric uncertainty in the robot dynamics and extend in this direction the current state of the art in motion planning and collision avoidance*.

### 3.2. Follower's control scheme

It should be noticed that the followers are not aware of either the object's desired trajectory or the obstacles in the workspace. However, even though explicit communication between the leader and the followers is not permitted, the followers will estimate the object's desired trajectory profile by ${\hat{P}}_{O}^{d_{i}}\left(t\right)$, *i* = 1, …, *N* via their own state measurements by adopting a prescribed performance estimator. Hence, let us define the errors $e^{i}\left(t\right)=P_{O}\left(t\right)-{\hat{P}}_{O}^{d_{i}}\left(t\right)\in {ℜ}^{6}$, *i* = 1, …, *N*. The expression of prescribed performance for each element of $e^{i}\left(t\right)={\left[e_{1}^{i}\left(t\right),\ldots ,e_{6}^{i}\left(t\right)\right]}^{T}$, *i* = 1, …, *N* is given by the following inequalities:

(9) $$-\rho_{j}^{i}\left(t\right)<e_{j}^{i}\left(t\right)<\rho_{j}^{i}\left(t\right)\text{, }j=1,\ldots ,6\text{ and }i=1,\ldots ,N$$

for all *t* ≥ 0, where $\rho_{j}^{i}\left(t\right)$, *j* = 1, …, 6 and *i* = 1, …, *N* denote the corresponding performance functions. A candidate exponential performance function could be:

$$\rho_{j}^{i}(t)=(\rho_{j,0}^{i}-\rho_{j,\infty }^{i})e^{-\lambda t}+\rho_{j,\infty }^{i}\text{, }i=1,\ldots ,$$

where the constant λ dictates the exponential convergence rate, $\rho_{j,\infty }^{i}$, *i* = 1, …, *N* denote the ultimate bounds and $\rho_{j,0}^{i}$ are chosen to satisfy $\rho_{j,0}^{i}>|e_{j}^{i}\left(0)\right|$, *i* = 1, …, *N*. Hence, following the prescribed performance control technique (Bechlioulis and Rovithakis, 2011), the estimation law is designed as follows:

(10) $${\dot{\hat{P}}}_{O_{j}}^{d_{i}}=k_{j}^{i}\ln\left(\frac{1+\frac{e_{j}^{i}\left(t\right)}{\rho_{j}^{i}\left(t\right)}}{1-\frac{e_{j}^{i}\left(t\right)}{\rho_{j}^{i}\left(t\right)}}\right),k_{j}^{i}>\text{0}$$

from which the followers' estimate ${\hat{P}}_{O}^{d_{i}}\left(t\right)=\left[{\hat{P}}_{O_{1}}^{d_{i}}\left(t\right),\ldots ,{\hat{P}}_{O_{6}}^{d_{i}}\left(t\right)\right]$, *i* = 1, …, *N* is calculated via a simple integration. Moreover, differentiating (10) with respect to time, we acquire the desired acceleration signal:

(11) $${\ddot{\hat{P}}}_{O_{j}}^{d_{i}}=\frac{2k_{j}^{i}}{1-{\left(\frac{e_{j}^{i}\left(t\right)}{\rho_{j}^{i}\left(t\right)}\right)}^{2}}\frac{{\dot{e}}_{j}^{i}\left(t\right)\rho_{j}^{i}\left(t\right)-e_{j}^{i}\left(t\right){\dot{\rho }}_{j}^{i}\left(t\right)}{{\left(\rho_{j}^{i}\left(t\right)\right)}^{2}}$$

employing only the velocity *Ṗ*_*O*_(*t*) of the object, which can be easily calculated via (2), and not its acceleration which is unmeasurable.

Lemma 1. *Consider the errors* $e^{i}\left(t\right)=P_{O}\left(t\right)-{\hat{P}}_{O}^{d_{i}}\left(t\right)={\left[e_{1}^{i}\left(t\right),\ldots ,e_{6}^{i}\left(t\right)\right]}^{T}$*, i* = 1, …, *N where P*_*O*_(*t*) *and* ${\hat{P}}_{O}^{d_{i}}\left(t\right)$, *i* = 1, …, *N* *denote the object's actual position/orientation and the estimation of the object's desired trajectory profile at the followers' side respectively. Given the leader's control scheme (8) as well as the appropriately selected performance functions* $\rho_{j}^{i}\left(t\right)$, *j* = 1, …, 6 *and* *i* = 1, …, *N* *satisfying* ${|e}_{j}^{i}\left(0)\right|<\rho_{j}^{i}\left(0\right)$, *j* = 1, …, 6 *and* *i* = 1, …, *N* *and incorporating the desired transient and steady state performance specifications, the estimation law (10) guarantees that* ${|e}_{j}^{i}\left(t)\right|<\rho_{j}^{i}\left(t\right)\text{,}j=1,\ldots ,6$ *and* *i* = 1, …, *N for all t* ≥ 0 *as well as that* ${\hat{P}}_{O}^{d_{i}}\left(t\right)$, ${\dot{\hat{P}}}_{O}^{d_{i}}\left(t\right)$ and ${\ddot{\hat{P}}}_{O}^{d_{i}}\left(t\right)$, *i* = 1, …, *N* *remain bounded*.

*Proof:* The proof follows identically for each element of *e*^*i*^(*t*), *i* = 1, …, *N*. Hence, let us first define the normalized errors with respect to the performance specifications encapsulated by the corresponding performance functions $\rho_{j}^{i}\left(t\right)$, as:

(12) $$\xi_{j}^{i}=\frac{e_{j}^{i}\left(t\right)}{\rho_{j}^{i}\left(t\right)}\text{, }j=1,\ldots ,6\text{ and }i=1,\ldots ,N\text{.}$$

The estimation law (10) may be rewritten as a function of the normalized error $\xi_{j}^{i}$ as follows:

(13) $${\dot{\hat{P}}}_{O_{j}}^{d_{i}}=k_{j}^{i}\ln\left(\frac{1+\xi_{j}^{i}}{1-\xi_{j}^{i}}\right)\text{, }j=1,\ldots ,6\text{ and }i=1,\ldots ,N\text{.}$$

Hence, differentiating $\xi_{j}^{i}$ with respect to time and substituting (13), we obtain:

(14) $${\dot{\xi }}_{j}^{i}=h_{j}^{i}\left(t,\xi_{j}^{i}\right)≜\frac{{\dot{P}}_{O_{j}}\left(t\right)-k_{j}^{i}\ln\left(\frac{1+\xi_{j}^{i}}{1-\xi_{j}^{i}}\right)}{\rho_{j}^{i}\left(t\right)}-\xi_{j}^{i}\frac{{\dot{\rho }}_{j}^{i}\left(t\right)}{\rho_{j}^{i}\left(t\right)}\text{.}$$

We also define the non-empty and open set $\Omega_{\xi_{j}^{i}}=\left(-1,1\right)$. In the sequel, we shall prove that $\xi_{j}^{i}\left(t\right)$ never escapes a compact subset of $\Omega_{\xi_{j}^{i}}$, thus meeting the performance bounds (9). The following proof is divided in two phases. First, we analyze the solution of the normalized errors and show that a maximal solution exists, such that $\xi_{j}^{i}\left(t\right)\in \Omega_{\xi_{j}}$ ∀*t* ∈ [0, τ_max_), whereas subsequently, via standard Lyapunov arguments, we prove by contradiction that τ_max_ is extended to ∞ and consequently that the errors $e_{i}^{j}\left(t\right)$ evolve strictly within the performance envelope described in (9).

*Phase A:* Since ${|e}_{j}^{i}\left(0)\right|<\rho_{j}^{i}\left(0\right)$, we conclude that $\xi_{j}^{i}\left(0\right)\in \Omega_{\xi_{j}^{i}}$. Moreover, owing to the smoothness of the object's trajectory and the proposed estimation scheme (10) over $\Omega_{\xi_{j}^{i}}$, the function $h_{j}^{i}\left(t,\xi_{j}^{i}\right)$ is continuous for all *t* ≥ 0 and $\xi_{j}^{i}\in \Omega_{\xi_{j}^{i}}$. Therefore, by Theorem 54 (pp.476) in Sontag (1998), a maximal solution $\xi_{j}^{i}\left(t\right)$ of (14) exists for the time interval [0, τ_max_) such that $\xi_{j}^{i}\left(t\right)\in \Omega_{\xi_{j}^{i}}$, ∀*t* ∈ [0, τ_max_).

*Phase B:* Notice that the transformed error signal:

(15) $$\varepsilon_{j}^{i}\left(t\right)=\ln\left(\frac{1+\xi_{j}^{i}\left(t\right)}{1-\xi_{j}^{i}\left(t\right)}\right)$$

is well defined for all *t* ∈ [0, τ_max_). Hence, consider the positive definite and radially unbounded function $V_{j}^{i}=\frac{1}{2}{\left(\varepsilon_{j}^{i}\right)}^{2}$. Differentiating with respect to time and substituting (14), we obtain:

$${\dot{V}}_{j}^{i}=\frac{2\varepsilon_{j}^{i}}{\left(1-{\left(\xi_{j}^{i}\right)}^{2}\right)\rho_{j}^{i}\left(t\right)}\left({\dot{P}}_{O_{j}}\left(t\right)-k_{j}^{i}\varepsilon_{j}^{i}-\xi_{j}^{i}{\dot{\rho }}_{j}^{i}\left(t\right)\right).$$

Since *Ṗ*_*O*_*j*__(*t*), *j* = 1, …, 6 was proven bounded in Theorem 1 for all *t* ≥ 0, and ${\dot{\rho }}_{j}^{i}\left(t\right)$ are bounded by construction, we conclude that:

$$\left|{\dot{P}}_{O_{j}}\left(t\right)+\xi_{j}^{i}{\dot{\rho }}_{j}^{i}\left(t\right)\right|\leq {\bar{d}}_{j}^{i},\;\forall t\in \left[0,\tau_{\max}\right)$$

for an unknown positive constant ${\bar{d}}_{j}^{i}$. Moreover, $\frac{1}{1-{\left(\xi_{j}^{i}\right)}^{2}}>1$, $\forall \xi_{j}^{i}\in \Omega_{\xi_{j}^{i}}$ and $\rho_{j}^{i}\left(t\right)>0$ for all *t* ≥ 0. Hence, we conclude that ${\dot{V}}_{j}^{i}<0$ when ${|\varepsilon }_{j}^{i}\left(t)\right|>\frac{{\bar{d}}_{j}^{i}}{k_{j}^{i}}$ and consequently that:

(16) $$\left|\varepsilon_{j}^{i}\left(t\right)\right|\leq {\bar{\varepsilon }}_{j}^{i}≜\max\left\{\left|\varepsilon_{j}^{i}\left(0\right)\right|,\frac{{\bar{d}}_{j}^{i}}{k_{j}^{i}}\right\}\text{, }\forall t\in \left[0,\tau_{\max}\right)\text{.}$$

Thus, invoking the inverse of (15), we get:

(17) $$-1<\frac{e^{-{\bar{\varepsilon }}_{j}^{i}}-1}{e^{-{\bar{\varepsilon }}_{j}^{i}}+1}={\underline{\xi }}_{j}^{i}\leq \xi_{j}^{i}\left(t\right)\leq {\bar{\xi }}_{j}^{i}=\frac{e^{{\bar{\varepsilon }}_{j}^{i}}-1}{e^{{\bar{\varepsilon }}_{j}^{i}}+1}<1.$$

Therefore, $\xi_{j}^{i}\left(t\right)\in \Omega_{\xi_{j}^{i}}^{\prime }=\left[{\underline{\xi }}_{j}^{i},{\bar{\xi }}_{j}^{i}\right]\text{,}\forall t\in \left[0,\tau_{\max}\right)$, which is a non-empty and compact subset of $\Omega_{\xi_{j}^{i}}$. Consequently, assuming τ_max_ < ∞ and since $\Omega_{\xi_{j}^{i}}^{\prime }\subset \Omega_{\xi_{j}^{i}}$, Proposition C.3.6 (p. 481) in Sontag (1998) dictates the existence of a time instant $t^{\prime }\in \left[0,\tau_{\max}\right)$ such that $\xi_{j}^{i}\left(t^{\prime }\right)\notin \Omega_{\xi_{j}^{i}}^{\prime }$, which is a clear contradiction. Therefore, τ_max_ is extended to ∞. As a result, all closed loop signals remain bounded and moreover $\xi_{j}^{i}\left(t\right)\in \Omega_{\xi_{j}^{i}}^{\prime }\subset \Omega_{\xi_{j}^{i}}$, ∀*t* ≥ 0. Thus, from (12) and (17), we conclude that:

$$-\rho_{j}^{i}\left(t\right)<{\underline{\xi }}_{j}^{i}\rho_{j}^{i}\left(t\right)\leq e_{j}^{i}\left(t\right)\leq {\bar{\xi }}_{j}^{i}\rho_{j}^{i}\left(t\right)<\rho_{j}^{i}\left(t\right)\text{, }\forall t\geq 0.$$

Finally, invoking (9)–(11) as well as the boundedness of *P*_*O*_ (*t*) and *Ṗ*_*O*_ (*t*) from Theorem 1, we also deduce the boundedness of ${\hat{P}}_{O}^{d_{i}}\left(t\right)$, ${\dot{\hat{P}}}_{O}^{d_{i}}$ and ${\ddot{\hat{P}}}_{O}^{d_{i}}$, *i* = 1, …, *N* for all *t* ≥ 0, which completes the proof.

Remark 4. *The proposed estimation scheme is more robust against trajectory profiles with non-zero acceleration than previous results presented in Kosuge and Oosumi (1996); Kosuge et al. (1997a,b). In particular, our method guarantees bounded closed loop signals and practical asymptotic stabilization of the estimation errors. Moreover, the aforementioned ultimate bounds depend directly on the design parameters* $\rho_{j,\infty }^{i}$, *j* = 1, …, 6 *and* *i* = 1, …, *N* *of the performance functions* $\rho_{j}^{i}\left(t\right)$, *j* = 1, …, 6 *and* *i* = 1, …, *N* *which can be set arbitrarily small to a value reflecting the resolution of the measurement device, thus achieving practical convergence of the estimation errors to zero. Additionally, the transient response depends on the convergence rate of the performance functions* $\rho_{j}^{i}\left(t\right)$, *j* = 1, …, 6 *and* *i* = 1, …, *N* *that is directly affected by the parameter* λ.

Based on the aforementioned estimation of the object's desired trajectory profile ${\hat{P}}_{O}^{d_{i}}\left(t\right)$, ${\dot{\hat{P}}}_{O}^{d_{i}}\left(t\right)$, and ${\ddot{\hat{P}}}_{O}^{d_{i}}\left(t\right)$, *i* = 1, …, *N* we can easily derive the corresponding desired trajectory profile for the follower's end-effector:

(18) $$\begin{array}{l} {\dot{P}}_{F_{i}}^{d}\left(t\right)≜{\hat{J}}_{F_{i}O}\left(t\right){\dot{\hat{P}}}_{O}^{d_{i}}\left(t\right)\\ {\ddot{P}}_{F_{i}}^{d}\left(t\right)≜{\hat{J}}_{F_{i}O}\left(t\right){\ddot{\hat{P}}}_{O}^{d_{i}}\left(t\right)+{\dot{\hat{J}}}_{F_{i}O}\left(t\right){\dot{\hat{P}}}_{O}^{d_{i}}\left(t\right) \end{array}\}\text{, }i=1,\ldots ,N$$

Let us also define the position and velocity errors:

$$e_{P_{F_{i}}}\left(t\right)=P_{F_{i}}\left(t\right)-P_{F_{i}}^{d}\left(t\right)\text{, }e_{{\dot{P}}_{F_{i}}}\left(t\right)={\dot{P}}_{F_{i}}\left(t\right)-{\dot{P}}_{F_{i}}^{d}\left(t\right)$$

as well as the first order stable linear filters:

(19) $$S_{F_{i}}\left(e_{P_{F_{i}}},e_{{\dot{P}}_{F_{i}}}\right)=\left(\frac{d}{dt}+\Lambda \right)e_{P_{F_{i}}}\equiv e_{{\dot{P}}_{F_{i}}}+\Lambda e_{P_{F_{i}}}\text{, }\Lambda >0$$

where *S*_*F*_*i*__ and *e*_*P*_*F*_*i*___ can be considered as input and output respectively. Notice that the tracking control problem for the followers' end-effector is equivalent to driving $S_{F_{i}}\left(e_{P_{F_{i}}}\left(t\right),e_{{\dot{P}}_{F_{i}}}\left(t\right)\right)$ to the origin, since for *S*_*F*_*i*__ = **0**, (19) represents a set of stable linear differential equations whose unique solution is *e*_*P*_*F*_*i*___ = **0** and *e*_*Ṗ*_*F*_*i*___ = **0**. In the sequel, we propose an adaptive control scheme for the followers' end-effector that guarantees the asymptotic convergence of the position and velocity errors to the origin.

**Theorem 2**. *Consider the unknown dynamics of the followers (3), that obey the parametric property (4), as well as the desired trajectory profiles (18) and the error metrics* $S_{F_{i}}\left(e_{P_{F_{i}}},e_{{\dot{P}}_{F_{i}}}\right)$ *defined in (19). The adaptive control scheme:*

(20) $$\begin{array}{l} U_{F_{i}}=-F_{F_{i}}+Z_{F_{i}}^{T}\left(P_{F_{i}},{\dot{P}}_{F_{i}},{\dot{P}}_{F_{i}}^{r},{\ddot{P}}_{F_{i}}^{r}\right){\hat{\theta }}_{F_{i}}-K_{F_{i}}S_{F_{i}}\text{, }K_{F_{i}}>0\\ {\dot{\hat{\theta }}}_{F_{i}}=-\Gamma_{F_{i}}Z_{F_{i}}\left(P_{F_{i}},{\dot{P}}_{F_{i}},{\dot{P}}_{F_{i}}^{r},{\ddot{P}}_{F_{i}}^{r}\right)S_{F_{i}},\Gamma_{{\text{F}}_{\text{i}}}>\text{0} \end{array}$$

*where* ${Ṗ}_{F_{i}}^{r}={Ṗ}_{F_{i}}^{d}\left(t\right)-\Lambda \left(P_{F_{i}}\left(t\right)-P_{F_{i}}^{d}\left(t\right)\right)$, ${\ddot{P}}_{F_{i}}^{r}={\ddot{P}}_{F_{i}}^{d}\left(t\right)-\Lambda \left({Ṗ}_{F_{i}}\left(t\right)-{Ṗ}_{F_{i}}^{d}\left(t\right)\right)$ *and* ${\hat{\theta }}_{F_{i}}$ *denotes the estimate of the unknown dynamic parameters θ_F_i__ of the followers' model, guarantees the asymptotic convergence of the position and velocity errors e_P_F_i___*(*t*), *e*_*Ṗ*_*F*_*i*___(*t*) *to the origin*.

*Proof:* The proof follows identical arguments for each follower *i* ∈ {1, …, *N*}. Hence, consider the positive definite function:

$$V_{F_{i}}=\frac{1}{2}S_{F_{i}}^{T}M_{F_{i}}\left(P_{F_{i}}\right)S_{F_{i}}+\frac{1}{2}{\tilde{\theta }}_{F_{i}}^{T}\Gamma_{F_{i}}^{-1}{\tilde{\theta }}_{F_{i}}\text{, }$$

where *M*_*F*_*i*__(*P*_*F*_*i*__) is the positive definite inertial matrix and ${\tilde{\theta }}_{F_{i}}={\hat{\theta }}_{F_{i}}-\theta_{F_{i}}$ denotes the parametric errors. Differentiating with respect to time and substituting the dynamics (3), we obtain:

$$\begin{array}{l} {\dot{V}}_{F_{i}}=S_{F_{i}}^{T}(U_{F_{i}}+F_{F_{i}}-M_{F_{i}}\left(P_{F_{i}}\right){\ddot{P}}_{F_{i}}^{r}-C_{F_{i}}\left(P_{F_{i}},{\dot{P}}_{F_{i}}\right){\dot{P}}_{F_{i}}\\ \;-D_{F_{i}}\left(P_{F_{i}},{\dot{P}}_{F_{i}}\right)-G_{F_{i}}\left(P_{F_{i}}\right))+\frac{1}{2}S_{F_{i}}^{T}{\dot{M}}_{F_{i}}\left(P_{F_{i}}\right)S_{F_{i}}\\ \;+{\tilde{\theta }}_{F_{i}}\Gamma_{F_{i}}^{-1}{\dot{\hat{\theta }}}_{F_{i}}\text{.} \end{array}$$

Adding and subtracting the term $S_{F_{i}}^{T}C_{F_{i}}\left(P_{F_{i}},{Ṗ}_{F_{i}}\right){Ṗ}_{F_{i}}^{r}$ yields:

$$\begin{array}{l} {\dot{V}}_{F_{i}}=S_{F_{i}}^{T}(U_{F_{i}}+F_{F_{i}}-M_{F_{i}}\left(P_{F_{i}}\right){\ddot{P}}_{F_{i}}^{r}-C_{F_{i}}\left(P_{F_{i}},{\dot{P}}_{F_{i}}\right){\dot{P}}_{F_{i}}^{r}\\ \;-D_{F_{i}}\left(P_{F_{i}},{\dot{P}}_{F_{i}}\right)-G_{F_{i}}\left(P_{F_{i}}\right))+{\tilde{\theta }}_{F_{i}}\Gamma_{F_{i}}^{-1}{\dot{\hat{\theta }}}_{F_{i}}\\ \;+\frac{1}{2}S_{F_{i}}^{T}\left({\dot{M}}_{F_{i}}\left(P_{F_{i}}\right)-2C_{F_{i}}\left(P_{F_{i}},{\dot{P}}_{F_{i}}\right)\right)S_{F_{i}}\text{.} \end{array}$$

Thus, invoking the parametric property (4) as well as the skew-symmetry of *Ṁ*_*F*_*i*__(*P*_*F*_*i*__)−2*C*_*F*_*i*__(*P*_*F*_*i*__, *Ṗ*_*F*_*i*__), we arrive at:

$${\dot{V}}_{F_{i}}=S_{F_{i}}^{T}\left(U_{F_{i}}+F_{F_{i}}-Z_{F_{i}}^{T}\left(P_{F_{i}},{\dot{P}}_{F_{i}},{\dot{P}}_{F_{i}}^{r},{\ddot{P}}_{F_{i}}^{r}\right)\theta_{F_{i}}\right)+{\tilde{\theta }}_{F_{i}}\Gamma_{F_{i}}^{-1}{\dot{\hat{\theta }}}_{F_{i}}\text{.}$$

Hence, substituting the control scheme (20), we get:

$${\dot{V}}_{F_{i}}=-S_{F_{i}}^{T}K_{F_{i}}S_{F_{i}}\leq 0$$

from which we may conclude the boundedness of *S*_*F*_*i*__ and ${\tilde{\theta }}_{F_{i}}$. Finally, employing Barbalat's Lemma, we may easily deduce that $\lim_{t\to \infty }S_{F_{i}}\left(t\right)=0$ and consequently the asymptotic convergence of the position and velocity errors *e*_*P*_*F*_*i*___(*t*), *e*_*Ṗ*_*F*_*i*___(*t*) to the origin, which completes the proof.     □

**Figure 4:**
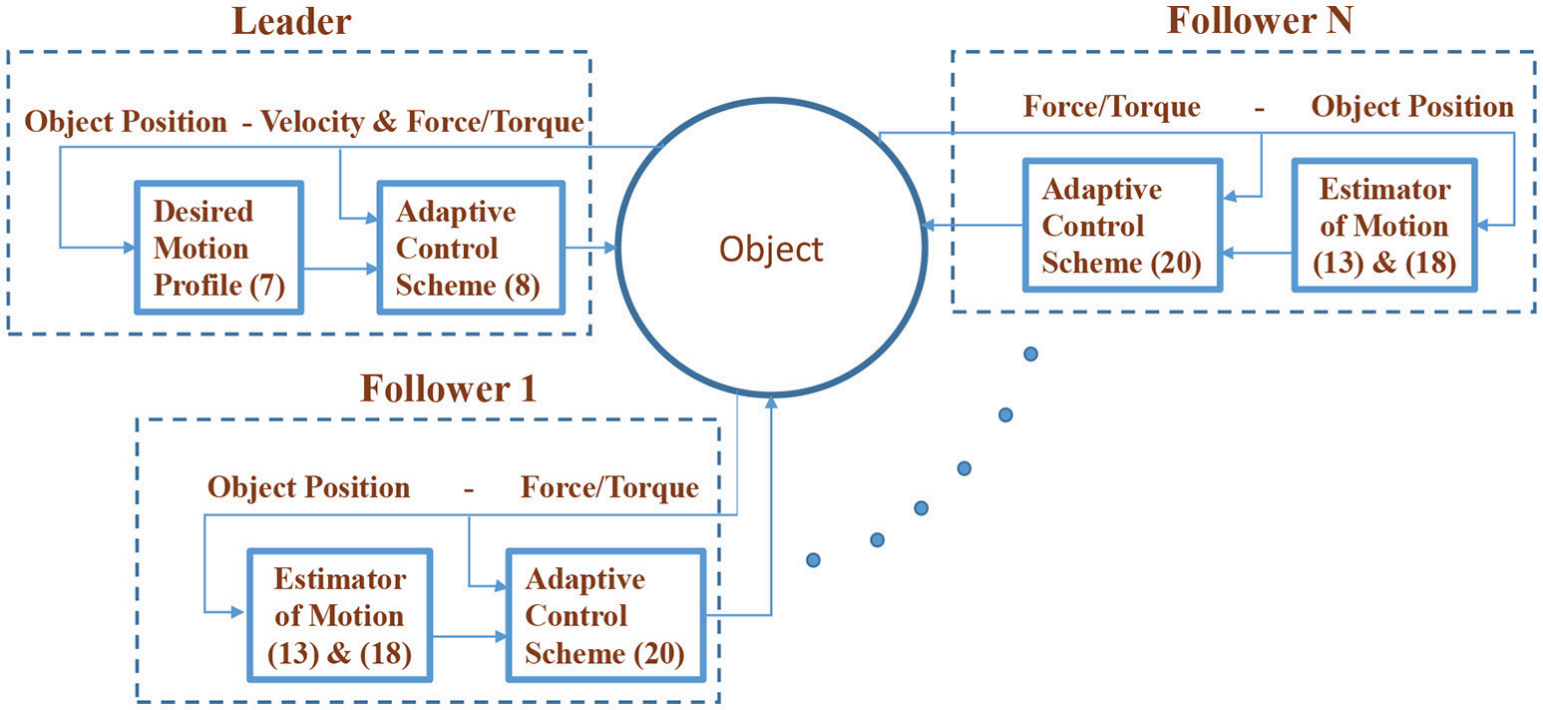
The overall control architecture.

Remark 5. *The proposed approach does not utilize any explicit on-line communication. The only information needed on-line to implement the developed control schemes concerns measurements acquired exclusively by each robot's sensor suite (i.e., force, position and velocity). Moreover, it is robust against parametric uncertainties in the robot dynamics. Further reinforcement of the closed loop robustness against model uncertainties could be achieved by introducing the σ-modification or deadzone techniques in the adaptive law (20), in order to handle the parameter drift issue. The overall control architecture is illustrated in Figure 4*.

## 4. Results

### 4.1. Simulation scenario A

**Figure 5:**
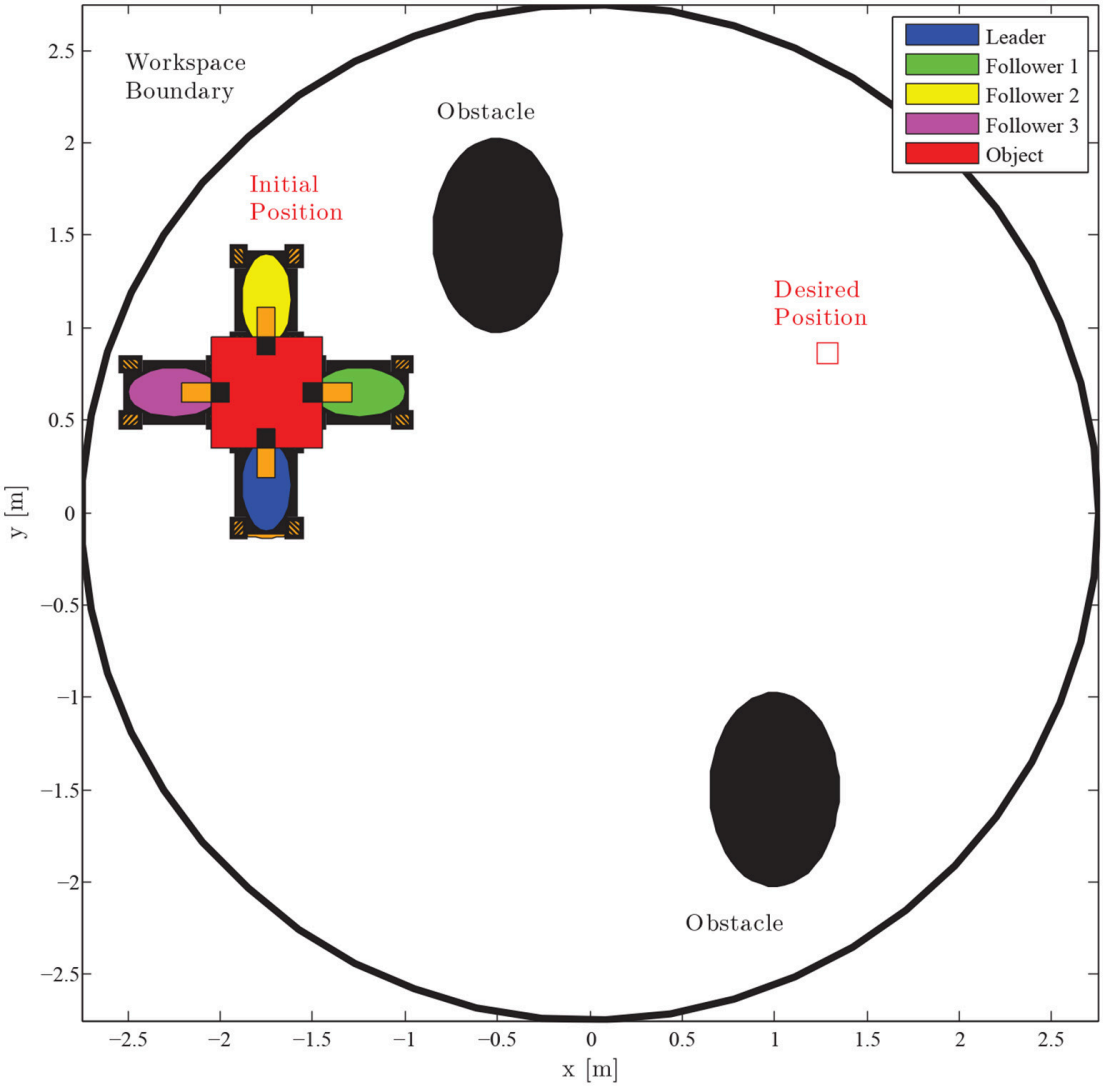
Four mobile manipulators handling a rigidly grasped object in a constrained workspace with static obstacles. Only the leader is aware of the object's desired configuration and the obstacles' position in the workspace.

We consider a scenario that involves the planar motion of four mobile manipulators in a leader-follower scheme, handling a rigidly grasped object in a constrained workspace with static obstacles (see Figure 5). The body frame of the object and the frame attached at the leader's end-effector are set aligned, while the followers's frame obtains a relative yaw angle of π/2 rad counter-clockwise. The leader is aware of the obstacles' position in the workspace and is assigned the desired object's configuration, whereas the followers estimate the object trajectory via the proposed algorithm (10), by simply observing the motion of the object and without communicating explicitly with the leader. Apparently, the overall formation has to transverse the obstacles in order to arrive at the desired configuration. The control gains were selected as follows: *k* = 2.15, *k*_*NF*_ = 0.8, *K*_*L*_ = 3**I**_2×2_, Λ = 3**I**_2×2_, *K*_*F*_ = 2**I**_2×2_. Additionally, since the robots' mass (i.e., *m*_*L*_ = *m*_*F*_ = 2.5 kgr) was considered unknown, the adaptive laws (8) and (20) were adopted to provide their estimates with control gains Γ_*L*_ = 0.1 and Γ_*F*_ = 0.15. Finally, the parameters of the proposed estimator were chosen as $k_{j}^{i}=1$, $\rho_{j}^{i}\left(t\right)=\left(2{|e}_{j}^{i}\left(0)\right|+0.1\right)e^{-3t}+0.05$, *j* ∈ {*x, y*} and *i* = 1, 2, 3.

**Figure 6:**
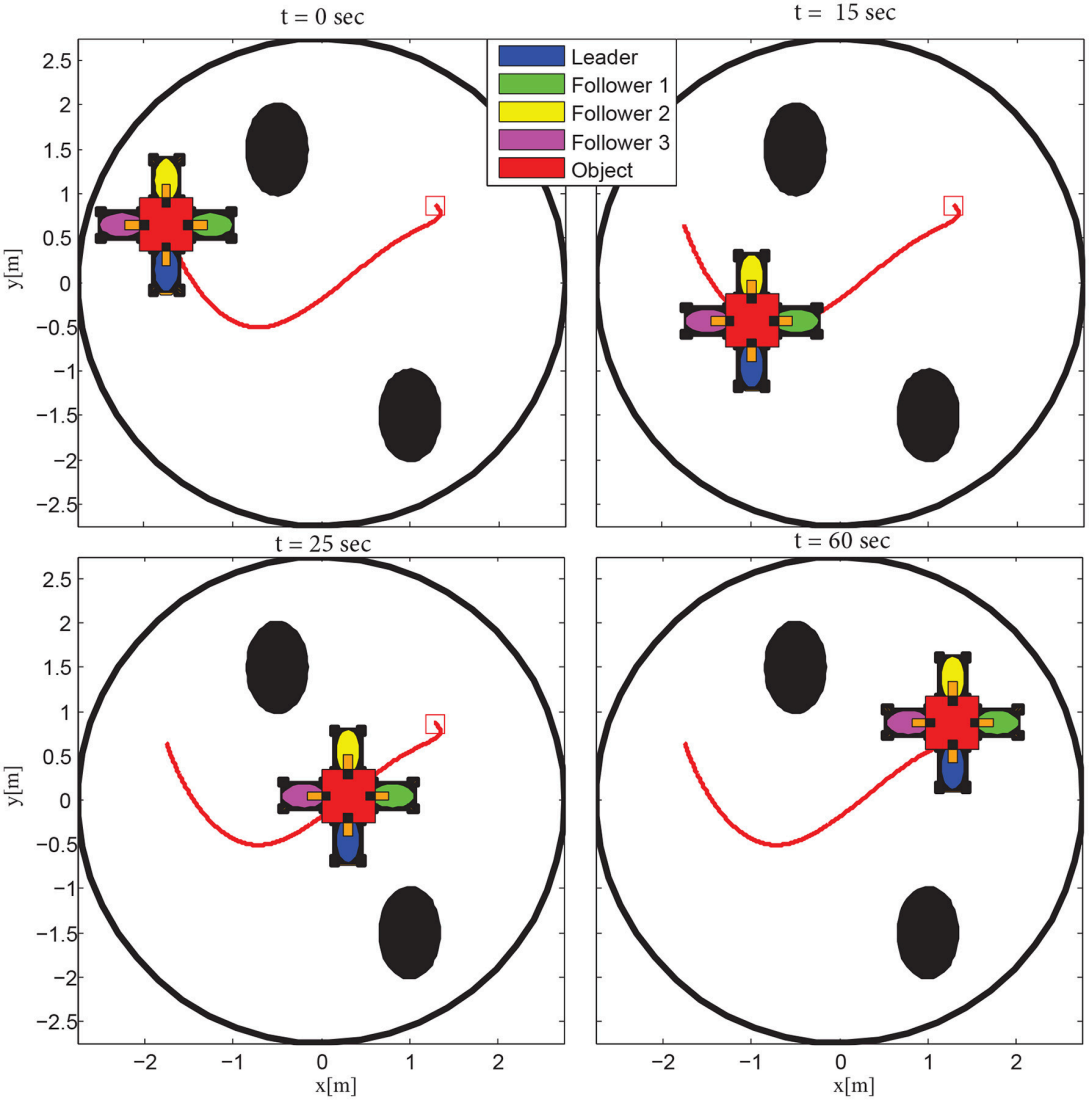
The evolution of the proposed methodology in four consecutive time instants.

**Figure 7:**
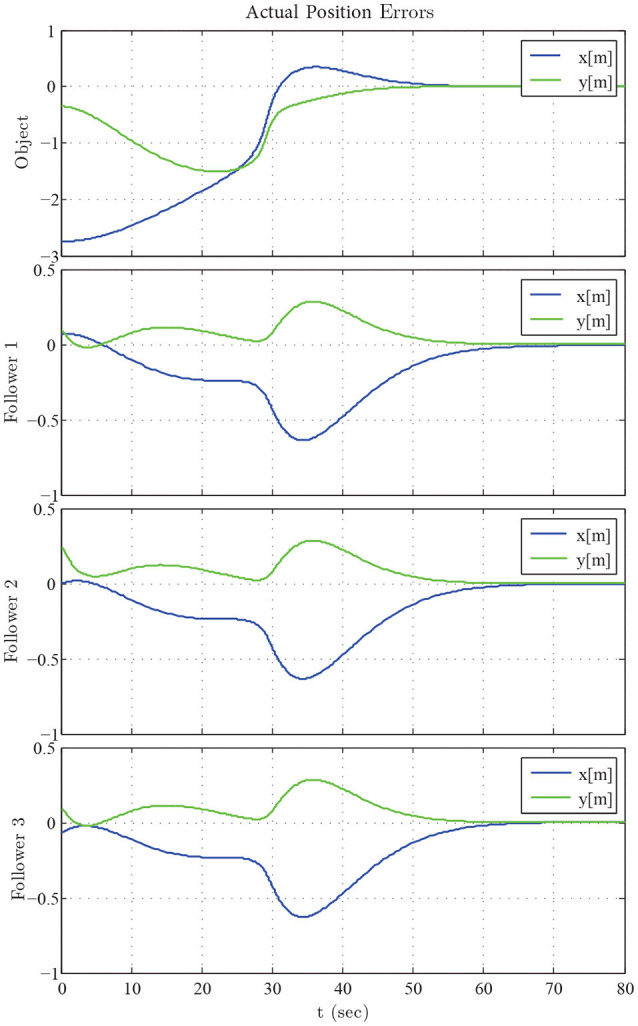
The actual position errors to the desired configuration.

**Figure 8:**
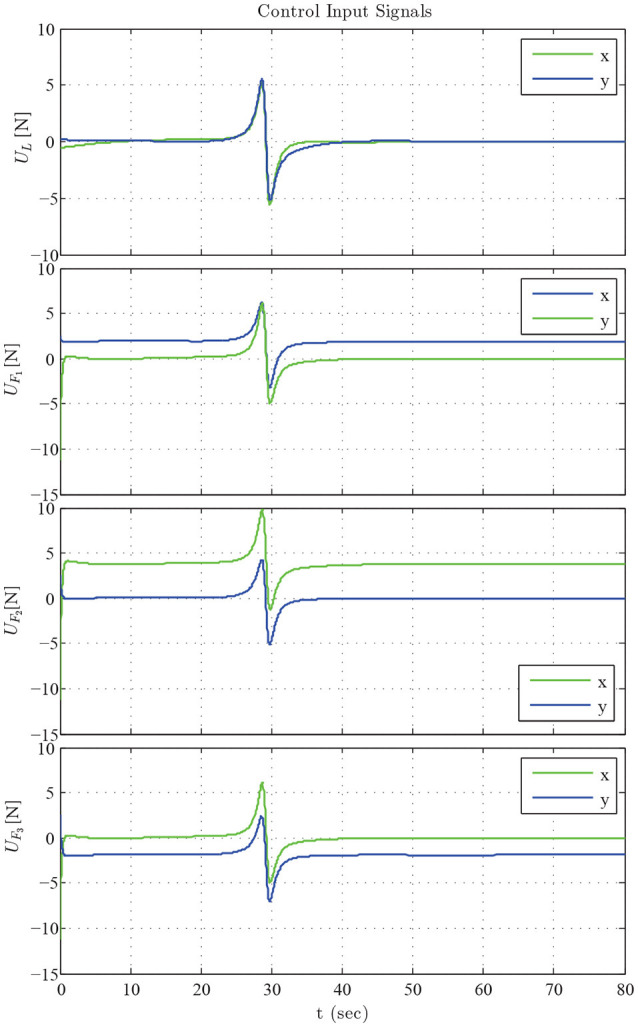
The control input signals *U*_*L*_ and *U*_*F*_*i*__, *i* = 1, 2, 3.

The results are given in Figures 6–8. More specifically, four consecutive instantiations of the simulated control algorithm are depicted in Figure 6. Notice that the overall formation maneuvers between the obstacles toward the desired configuration, which is attained in 60 s. The position errors with respect to the desired configuration of each robot are illustrated in Figure 7. Finally, the required control inputs for all agents are given in Figure 8. It should be stressed that reasonable overshoot (i.e., less than five times the effort requested at the steady state, which is acceptable from a practical point of view) on the control signals occurs initially as well around the middle of the simulation, where the formation transverses the obstacles and reaches the desired configuration.

### 4.2. Simulation scenario B

**Figure 9:**
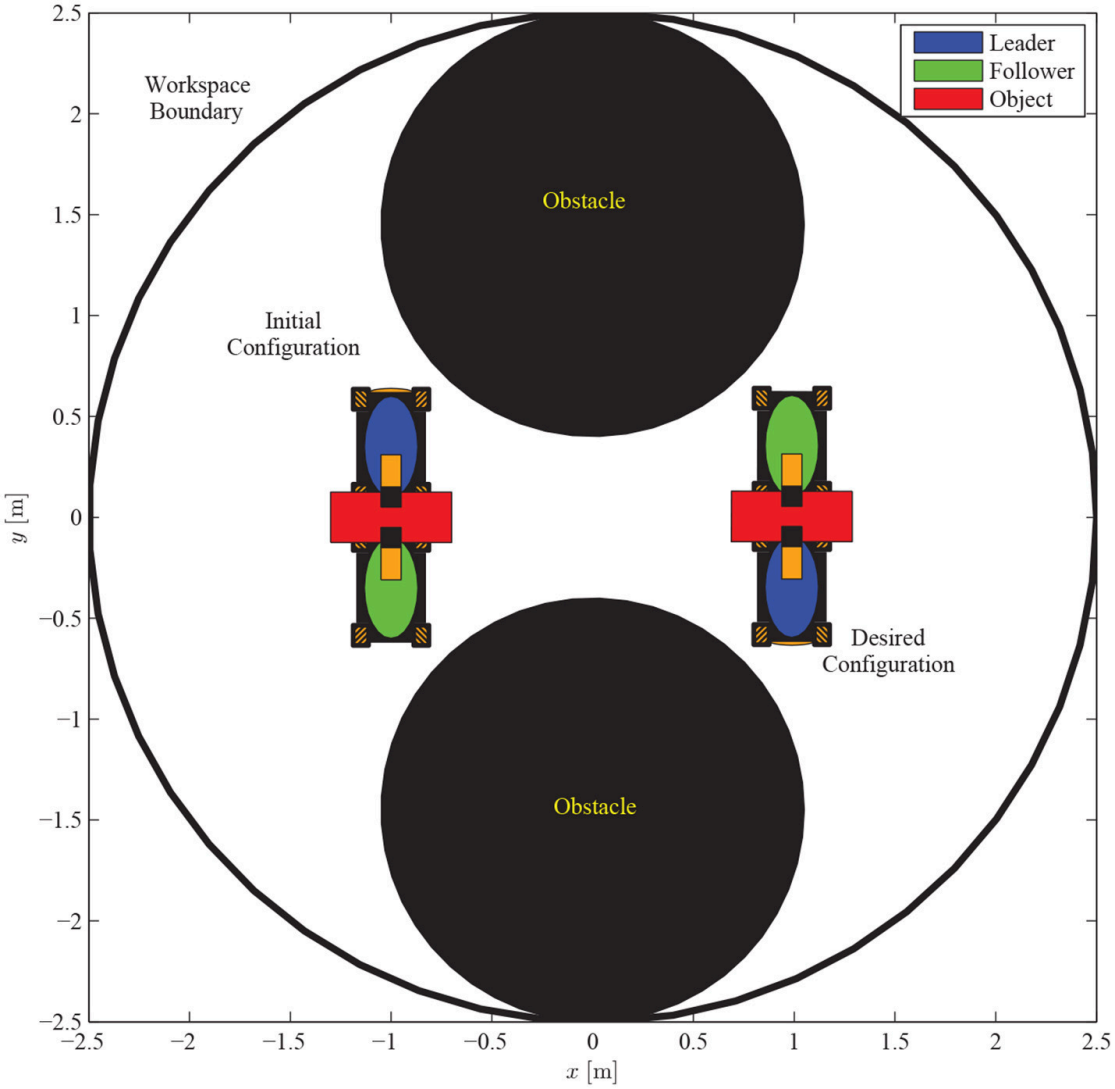
Two mobile manipulators handling a rigidly grasped object in a constrained workspace with static obstacles. Only the leader is aware of the object's desired configuration and the obstacles' position in the workspace.

**Figure 10:**
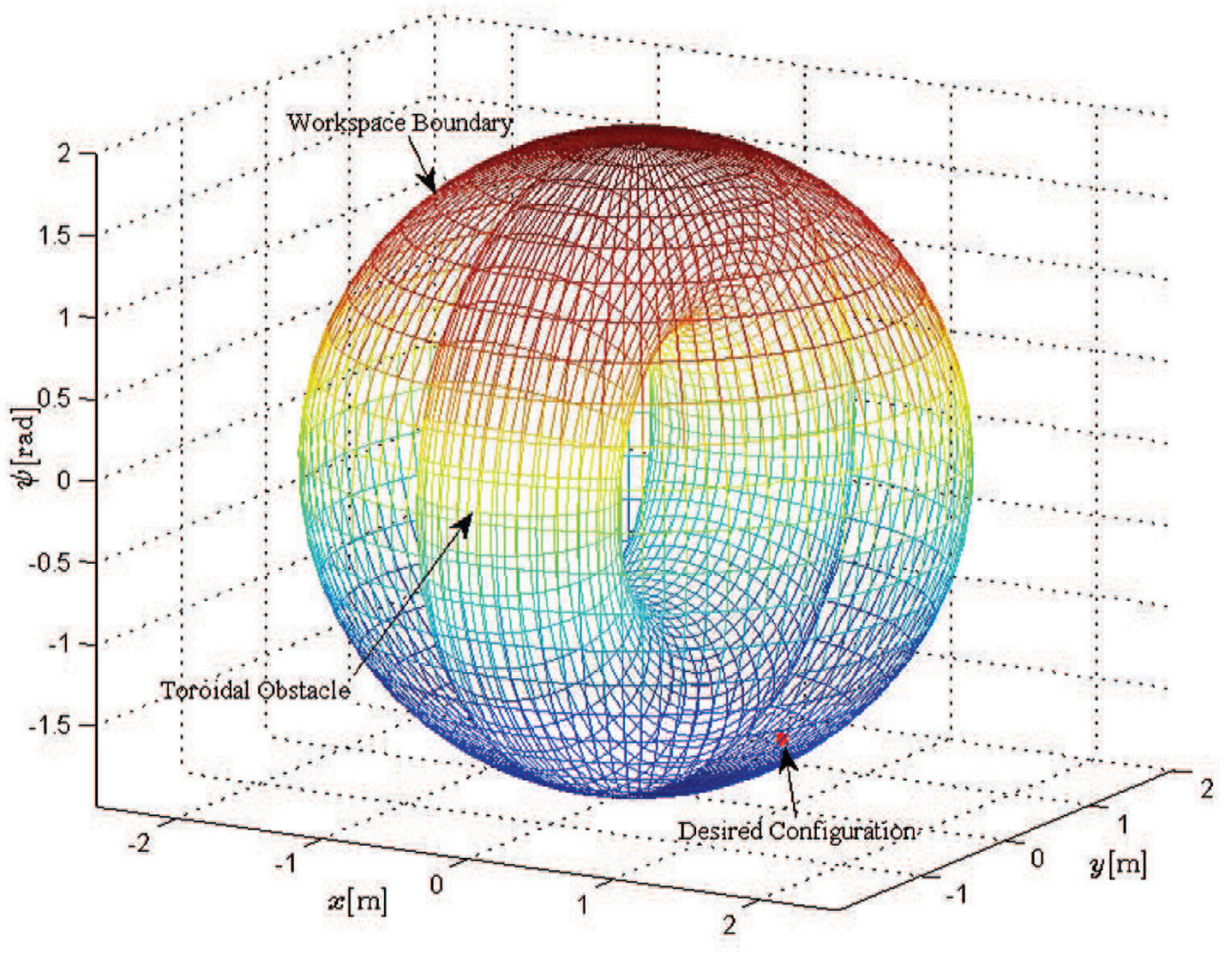
The feasible workspace for oriented planar motion of a long formation.

We consider a scenario involving oriented motion on a planar surface (i.e., the coordinates are *x*, *y* and the orientation ψ) with two mobile manipulators in a leader-follower scheme, handling a rigidly grasped object in a constrained workspace with static obstacles (see Figure 9). The body frame of the object and the leader's end-effector frame are set aligned, while the follower's frame has a relative yaw angle of π rad. The leader is aware of the obstacles' position in the workspace and is assigned the desired object's configuration, whereas the follower estimates the object's trajectory via the proposed algorithm (10), by simply observing the motion of the object and without communicating explicitly with the leader. Apparently, the overall formation has to be aligned with the *x*-axis in order to transverse the obstacles. In this respect, we constructed a navigation function in a 3D workspace (i.e., *x*, *y*, and ψ), by adopting a virtual toroidal obstacle (see Filippidis and Kyriakopoulos, 2012 for the safety and convergence properties) to model the aforementioned relation of position (*x, y*) with the orientation ψ, as depicted in Figure 10. Moreover, the control gains were selected as follows: *k* = 1.9, *k*_*NF*_ = 0.8, *K*_*L*_ = 3**I**_3×3_, Λ = 3**I**_3×3_, *K*_*F*_ = 3**I**_3×3_. Additionally, since the robots' mass (i.e., *m*_*L*_ = *m*_*F*_ = 2.5 kgr) was considered unknown, the adaptive laws (8) and (20) were adopted to provide their estimates with control gains Γ_*L*_ = 0.1 and Γ_*F*_ = 0.15. Finally, the parameters of the proposed estimator were chosen as *k*_*j*_ = 1, $\rho_{j}\left(t\right)=\left(2|e_{j}\left(0)\right|+0.1\right)e^{-3t}+0.05$, *j* ∈ {*x, y*, ψ}.

**Figure 11:**
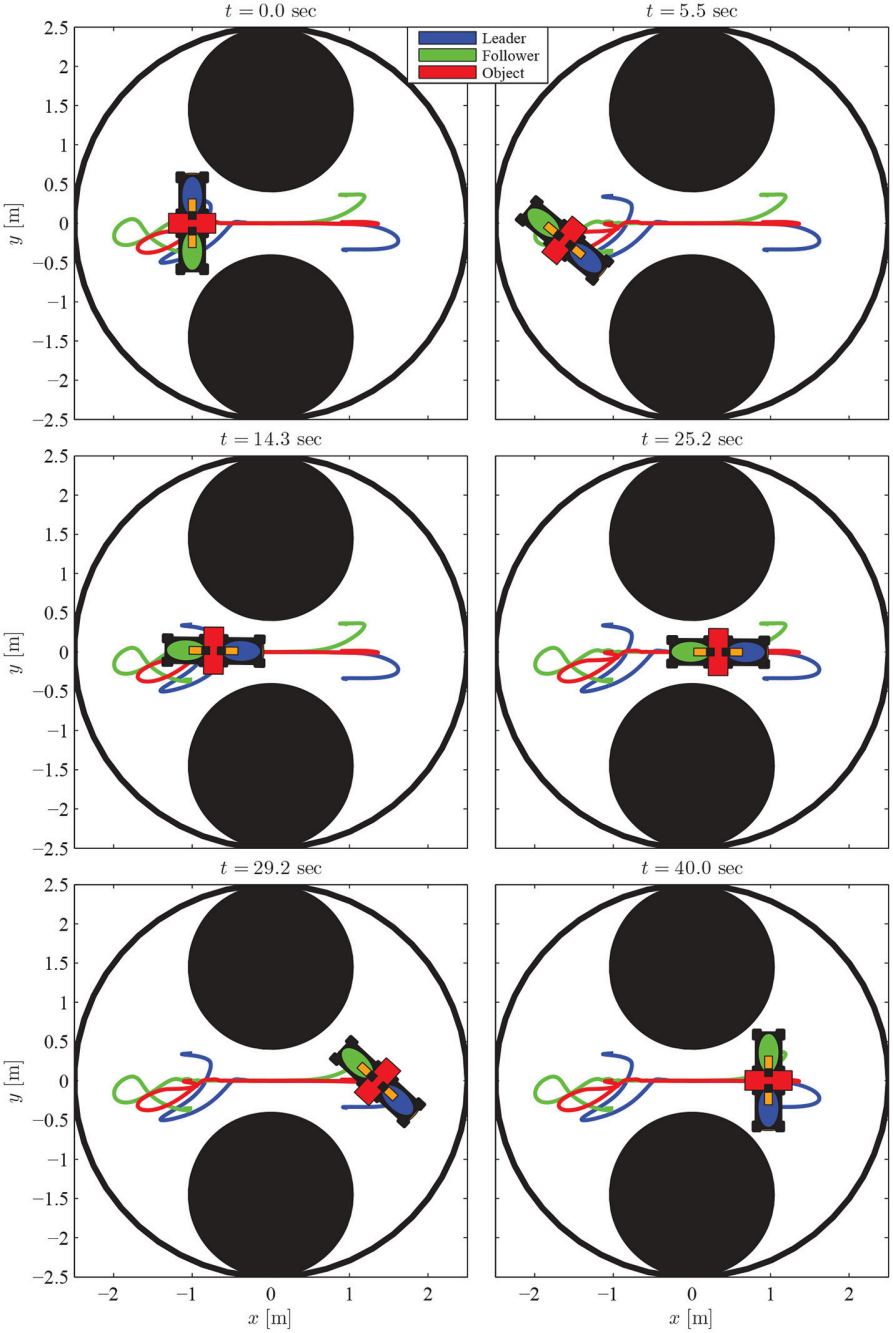
The evolution of the proposed methodology in six consecutive time instants.

**Figure 12:**
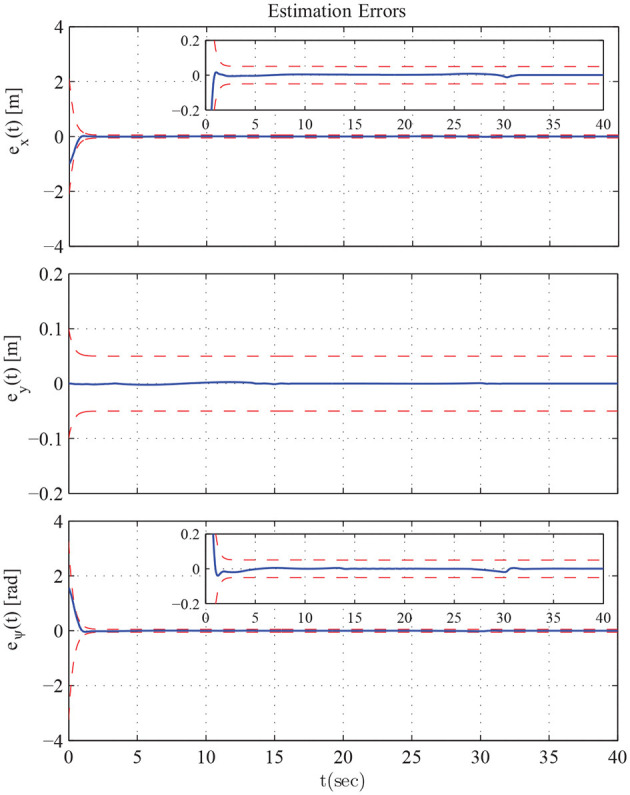
The estimation errors along with the performance bounds imposed by the proposed method.

**Figure 13:**
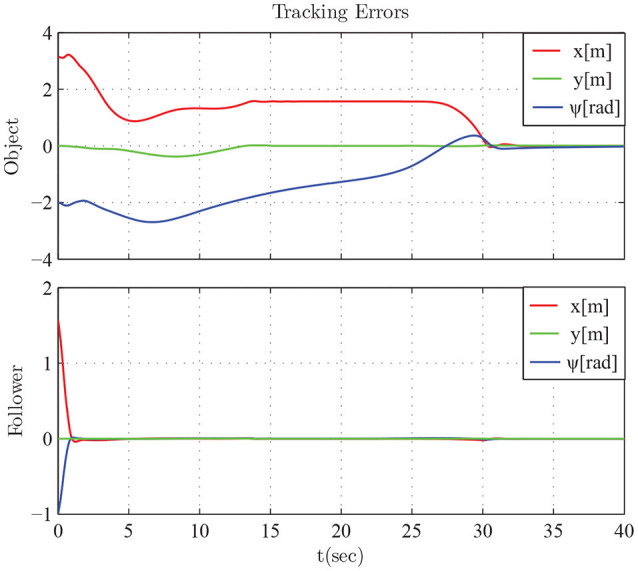
The tracking errors.

**Figure 14:**
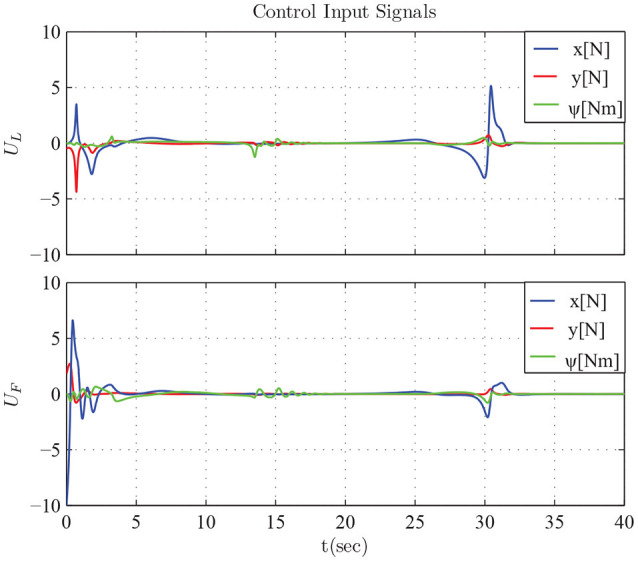
The control input signals *U*_*L*_ and *U*_*F*_.

**Figure 15:**
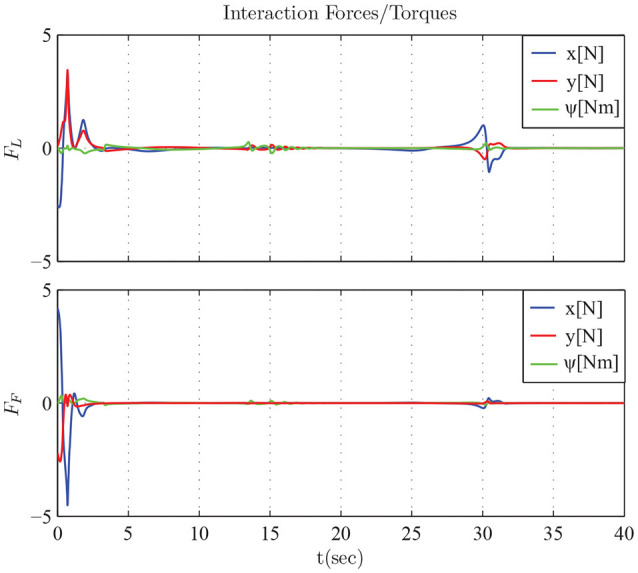
The interaction forces *F*_*L*_ and *F*_*F*_ exerted between the object and the robots.

The results are given in Figures 11–15. In particular, the evolution of the simulation of the proposed scheme for six consecutive time instants is illustrated in Figure 11. The estimation errors of the trajectory of the object are depicted in Figure 12 along with the performance bounds imposed by the appropriately selected performance functions. Notice that after a short transient, the estimation errors converge close to zero and are kept small afterwards. The tracking errors with respect to the desired object configuration and the estimated object trajectory by the follower are illustrated in Figure 13. It should be noted that the object arrives at its desired configuration in 30 sec via the appropriate motion planning executed by the leader. On the other hand, the follower tracks quickly the object's estimated trajectory and collaborates with the leader toward the successful fulfillment of the transportation task. Finally, the requested control inputs (i.e., forces along *x*, *y* and torque around *z*) as well as the interaction forces/torque between the robots and the commonly grasped object are depicted in Figures 12, 13. It should be stressed that the control effort and consequently the interaction forces/torque obtain high but reasonable values when the formation is maneuvering at the initial and final stage to transverse the obstacles and attain the desired orientation respectively.

## 5. Conclusions

This paper presented a leader-follower scheme for cooperative object transportation under implicit communication, thus avoiding completely tedious explicit on-line inter-robot communication. The leader that was aware of both the desired configuration of the object as well as of the obstacles' position in the workspace, aimed at navigating the overall formation toward the goal configuration while avoiding collisions with static obstacles. On the contrary, the followers adopted a prescribed performance estimator to evaluate the object's desired trajectory that were unaware of. We extended the related literature by: (i) combining innovatively Navigation Functions with adaptive control to deal with parametric uncertainty in the robot dynamics and (ii) robustifying the estimation process against any smooth and bounded object's desired trajectory profile. Future research efforts will be devoted toward extending the current methodologies for environments with dynamically moving obstacles (i.e., humans) via employing other types of implicit communication and relative sensing, acquired by onboard sensors such as cameras, range finders or laser scanners, that would increase the applicability of the proposed scheme. Moreover, considering uncertainties in the model of the grasped object and its geometry is also left open for future investigation. Finally, generalizing the results of this work into a scheme with multiple leaders would significantly increase the robustness against faults/failures and lead eventually in a fully decentralized approach with dynamic assignment of the leading roles among the team members, for which only lean explicit communication would be requested.

## Author contributions

All authors listed have made a substantial, direct and intellectual contribution to the work, and approved it for publication.

### Conflict of interest statement

The authors declare that the research was conducted in the absence of any commercial or financial relationships that could be construed as a potential conflict of interest.

## Footnotes

^1^Since the proposed approach is based solely on the relative information among the object and the robots, each robot may operate on its own inertial frame and calculate with respect to it all required information regarding the object, thus rendering the approach decentralized.

^2^Other geometrically more complex workspaces may also be considered by adopting proper topologically equivalent transformations presented in Rimon and Koditschek (1992) and Vlantis et al. (2018).

^3^Except from a set of measure zero, see Koditschek and Rimon (1990).

^4^As a sum of bounded terms.
